# Effect of Capsaicin and Dihydrocapsaicin in Capsicum on Myofibrillar Protein in Duck Meat

**DOI:** 10.3390/foods12193532

**Published:** 2023-09-22

**Authors:** Wei Sun, Wenjie He, Danjun Guo, Wei Xu

**Affiliations:** 1 College of Food Science and Engineering, Wuhan Polytechnic University, Wuhan 430023, China; sunshuaiwen@163.com (W.S.); hhh19970401@163.com (W.H.); xuwei1216@163.com (W.X.); 2 Key Laboratory for Deep Processing of Major Grain and Oil (Wuhan Polytechnic University), Ministry of Education, Wuhan 430023, China; 3 Hubei Key Laboratory for Processing and Transformation of Agricultural Products, Wuhan 430023, China

**Keywords:** capsaicin, dihydrocapsaicin, myofibrillar protein, properties

## Abstract

Spice and its extracts have gained widespread utilization as natural and eco-friendly additives, imparting enhancements in flavor, color, and antioxidative attributes to meat-based products. This work aims to study the effect mechanism of capsaicin (CA) and dihydrocapsaicin (DI) in capsicum (chili pepper) on the structure and function of myofibrillar proteins (MPs) in duck meat during thermal treatment. The results showed that at a CA–DI to MP ratio of 1:500 (g/g) following a 12 min heat treatment, the carbonyl content of MPs in duck meat decreased by 48.30%, and the sulfhydryl content increased by 53.42%. When the concentration was 1:500 (CA-DI, g/g) after 24 min of heat treatment, the •OH and DPPH radical scavenging rates were highest at 59.5% and 94.0%, respectively. And the initial denaturation temperature of MPs was the highest at 96.62 °C, and the thermal absorption was lowest at 200.24 J g^−1^. At the parameter, the smallest particle size and size distribution range of MP were 190 nm (9.51%). Furthermore, the interplay between CA–DI and MPs contributed to a reduction in the protein particle size and intrinsic fluorescence. In summary, the combination of CA–DI and MPs played a crucial role in inducing protein unfolding and disintegration.

## 1. Introduction

Duck meat, which is well known for its distinct flavor, aromatic nuances, elevated protein content, large polyunsaturated fatty acid composition, and economic affordability, comprises a substantial proportion of consumption patterns, particularly across Asia [1,2]. However, the nutritive value and functional attributes of duck meat are notably susceptible to compromise due to protein oxidation and lipid oxidative rancidity [3,4]. Heat treatment is one of the factors that causes protein oxidation, leading to a decline in the taste and nutrition of meat products. The dominant constituents within a muscular protein, matrices myofibrillar proteins (MPs,) have been the subject of comprehensive investigations concerning protein oxidation across diverse meat varieties [5]. During the oxidative process, a significant outcome is the emergence of protein cross-linking, which exerts discernible ramifications on the gelling properties, emulsifying propensities, and water-holding competence that are intrinsic to myofibrillar proteins. This, in turn, precipitates a decline in intact meat tenderness but may improve the texture of processed meat products, as signified by elevated shear forces [6,7,8]. The textural properties, coloration, aroma, and flavor further manifest within the context of myofibrillar protein oxidation [9,10]. The oxidative transformation of meat proteins engenders a cumulative depletion of specific amino acids, alongside substantial reconfigurations in the amino acid profile, consequently bearing implications for the availability of essential amino acids crucial for human nutrition [11,12]. Moreover, an array of investigations has attested to the compromised protein digestibility encountered within the human digestive milieu, attributable to the ramifications of protein oxidation [13,14,15]. By compounding these intricate considerations, the processing and digestive phases of muscle-derived comestibles concurrently give rise to the accrual of particular protein oxidation products, thereby conferring potential health hazards inherent to the toxicity of such entities [16].

Spices, botanical entities imbued with multifarious aromatic and gustatory attributes encompassing fragrant, pungent, numbing, bitter, and sweet nuances, have garnered increased attention as natural antioxidant compounds that may impact meat processing [17]. This paradigm shift toward incorporating an expanding repertoire of spices and their extracts in meat formulations aims to counteract protein oxidation while concurrently augmenting sensory dimensions encompassing color, flavor, aroma, and overall palatability [18]. The use of lemon grass (Cymbopogon citratus) and star anise (Illicium verum) extracts has been demonstrated to effectively scavenge DPPH and superoxide radicals within minced chicken meat matrices [19]. Similarly, Cao et al. studied the impact of yam saponin, hesperidin, and ginger extracts, and observed the pronounced enhancement of myofibrillar protein aggregation coupled with the augmented water-holding capacity of resultant gels [20]. Estevez et al. introduced rosemary essential oil as an augmentative agent in frankfurter formulation, delineating its role in ameliorating texture attributes, as characterized by the mitigation of *hardness*, *adhesiveness*, *gumminess*, and *chewiness*, alongside the regulation of *elasticity* loss during refrigeration [10]. The realm of spice exploration extends to antimicrobial potential, where extracts derived from Padang cassia, Chinese cassia, and clove at a concentration of 0.03% have demonstrated efficacy against foodborne pathogenic bacteria, thereby concomitantly influencing meat sensory attributes [21]. An overarching theme of these investigations is the inherent antibacterial capacities attributed to the phenolic compounds pervasive within diverse spices [22].

However, scientific scrutiny of the influence of capsicum (chili pepper) on myofibrillar proteins within duck meat remains relatively scant. Capsicum, imbued with connotations encompassing culinary and medicinal dimensions, serves as an effective approach within the antioxidant and antibacterial domains in animal feeding studies and pharmacological investigations [23,24]. In meat science, capsicum and its compounds are widely used in chicken, pork, and sausage to enhance the quality characteristics and inhibit protein oxidation in meat products [25,26,27]. Additionally, capsicum is an indispensable facet of the global culinary landscape, responsible for chromatic and gustatory harmonies across a myriad of cultural milieus. Some additional study is necessary into the effects of capsicum compounds on duck meat. Capsaicin (CA) and dihydrocapsaicin (DI) have emerged as the principal bioactive entities intrinsic to capsicum, distinguished by a CA to DI ratio ranging from 1.5 to 2.9 [28]. An experiment on the blending of duck myofibrillar protein isolate with CA–DI (at a 2:1, g/g ratio) could reduce the shear force within duck meat. The mechanism underpinning the interplay between CA–DI and myofibrillar proteins during thermal treatment remains unclear. The present research aims to unravel the intricate underpinnings of the impact of CA–DI on the structural moieties of myofibrillar proteins within duck meat by heat treatment and provide a theoretical framework that highlights the role of Capsicum in improving duck meat product properties during the processing.

## 2. Materials and Methods

### 2.1. Materials and Reagents

Frozen duck leg meat samples (male, postmortem time 24 h, frozen storage length 2 weeks at −20 °C, moisture 72.8%, protein 15.2%, and rude fat 7.5%) were sourced from Hubei Chia Tai Egg Industry Co. Ltd. (Shanghai, China). The CA (purity: HPLC ≥ 98%) and DI (purity: HPLC ≥ 98%) compounds were procured from Shanghai Tongwei Biotechnology Co. Ltd. (Shanghai, China). It is imperative to note that all chemicals and reagents employed in the experimental procedures were of analytical grade, unless specified otherwise.

### 2.2. Extraction of MPs

Duck MPs were prepared in accordance with the method outlined by Park et al. incorporating discerning modifications [12]. The duck meat was thawed at 4 °C for 24 h before analysis. First, duck meat was meticulously rinsed with saline solution (0.90%) and then desiccated using filter paper. The ensuing muscle was meticulously sectioned into fillets and then minced (Boxun YB-1000A, Shanghai, China) by grinding using a plate with 6 mm holes. The minced tissue was subsequently mixed with a pH 6.5 buffer solution, comprising constituents of 25 mmol/L KCl, 150 mmol/L NaCl, 4 mmol/L EDTA-2Na, 3 mmol/L MgCl_2_, and 1 mmol/L PMSF, maintaining a specific proportion of 1:10 (g/mL, *w*/*v*). The mixture was homogenized at 10,000 rpm for 30 s, (Xinzhi XHF-DY, Ningbo, China), followed by centrifugation at 2205 g and 4 °C for a duration of 30 min (Anting TGL16M, Shanghai, China). The supernatant obtained was discarded, and the precipitate was subjected to a wash sequence, repeated three times, employing a buffer solution composed of 50 mmol/L potassium chloride alongside 5 mmol/L β-mercaptoethanol. Finally, the resultant pellet underwent extraction via buffer solution, thereby culminating in the generation of a homogenized suspension constituting the MPs extract. The quantitative assessment of protein concentration was undertaken through the utilization of the Coomassie brilliant blue method [29], with the MP concentration ultimately standardized to 1.00 mg/mL to facilitate the objectives of this study.

### 2.3. MPs Sample Preparation

In accordance with established laboratory protocols, the extracted MPs were initially subjected to incubation within a water bath (40 °C) for a duration of 7 min. Subsequently, a composite solution comprising capsaicin (CA) and dihydrocapsaicin (DI) in a ratio of 2:1 (g/g) was introduced to the MPs matrix at ratios of 1:1000, 1:500, 1:250, and 3:500 (CA–DI to MPs, g/g). This amalgamation was then subjected to thermal treatment at 90 °C over a period of 36 min. At defined intervals of 12 min, corresponding to time points encompassing 12 min, 24 min, and 36 min, aliquots of the resultant mixed solution were used for subsequent analysis and detection. Notably, the binding modality between CA–DI and MPs is elucidated in the Appendix A.

### 2.4. Carbonyl Content

The quantification of carbonyl content was conducted utilizing a method described in [30]. A total of 2 mL aliquot of the extracted MPs sample solution was meticulously diluted using an equal volume of 2 mol/L hydrochloric acid (HCl) solution fortified with 10 mmol/L 2,4-dinitro-phenylhydrazine (DNPH). This composite mixture was incubated at a temperature of 25 °C for a stipulated duration of 1 h. After this incubation phase, an I confirm. additional 2 mL of 20% trichloroacetic acid (TCA) was introduced into the solution, which was consequently subjected to centrifugation at 353 g for a span of 5 min employing the Anting TGL16M centrifuge (Shanghai, China). The ensuing supernatant was selectively decanted, leaving behind the resultant precipitate. This precipitate was subsequently combined with 6 mL of a solution containing 6 mol/L guanidine hydrochloride, and this mixture was incubated in a water bath maintained at 37 °C for a duration of 15 min. Protein content was ascertained via UV2000 spectrophotometric analysis employing a wavelength of 280 nm. It is pertinent to underscore that the standard calibration curve was formulated using bovine serum protein solubilized within a solution of 6 mol/L guanidine hydrochloride. Concurrently, the quantitative assessment of carbonyl content was subjected to the measurement of absorbance at a wavelength of 370 nm, performed utilizing a Unico UV2000 spectrophotometer (Shanghai, China). To determine the carbonyl content, the molar absorbance coefficient of 22,000 M^−1^ × cm^−1^ was invoked, resulting in the expression of protein carbonyl content in units of nmol/mg protein. Analysis for each individual sample was repeated three times.

### 2.5. Free Sulfhydryl Content

The quantification of free hydroxyl content was conducted following a method that was modestly adapted [31]. For this experiment, a 2 mL aliquot of the extracted MPs sample solution was amalgamated with 4 mL of Tris buffer, along with an additional 1 mL of 10 mmol/L 5,5′-dithiobis-(2-nitrobenzoic acid) (DTNB), the latter of which was solubilized within a 0.1 mol/L Tris buffer at a pH of 8.0. To serve as a comparative reference, 5% sodium dodecyl sulfate (SDS) dissolved in 0.1 mol/L Tris buffer at a pH of 8.0 was employed in lieu of the conventional 1 mL diluent, assuming the role of the blank control. After incubation at 25 °C for 30 min, the absorbance of the resultant supernatant was ascertained at a wavelength of 412 nm. Quantitative evaluation of sulfhydryl content was conducted using the molecular absorbance coefficient, denoted as 13,600 M^−1^ × cm^−1^, which finally expressed the outcomes in units of nmol/mg. Analysis of each individual sample was performed three times to ensure robustness and reproducibility of results.

### 2.6. Surface Hydrophobicity

The evaluation of MP surface hydrophobicity was carried out by employing 1-benzenedioic acid-8-sulfonic acid (ANS) as the pivotal fluorescent probe, following an established methodology with minor refinements [32]. To initiate this assessment, a 2 mL aliquot of the sample solution was meticulously combined with 10 uL of 0.1 mol/L phosphate buffer, wherein the latter was supplemented with 8 mmol/L ANS and adjusted to a pH of 7.0. After this preparation, fluorescence intensity measurements were collected at an emission wavelength of 470 nm and an excitation wavelength of 390 nm utilizing a fluorescence spectrophotometer (Hitachi F-7000, Tokyo, Japan). The configured settings for the excitation and emission slit width were both set at 5 nm. The resultant fluorescence intensity data were subjected to a graphical representation, with protein concentration serving as the abscissa and the slope of the initial segment within this plot bestowed the surface hydrophobicity value of MPs. As a testament to rigorous methodology, each individual sample was subjected to a triad of analyses to ensure reliability and reproducibility.

### 2.7. Hydroxyl Radical Scavenging Activity

The assessment of hydroxyl radical scavenging activity was conducted employing the methodology outlined by Zhu et al. [33]. A total of 1 mL of each respective solution was mixed in a 10 mL centrifuge tube. The constituents included the specimen solution: a 6 mmol/L solution of salicylic acid in ethanol, a 6 mmol/L solution of ferrous sulphate, and a 6 mmol/L solution of hydrogen peroxide. After thorough mixing, the solution was maintained at a temperature of 37 °C for a duration of 30 min. The resultant absorbance was quantified, and the extent of hydroxyl radical scavenging was deduced in accordance with the subsequent mathematical expression. Each distinct sample was tested in triplicate to ensure statistical robustness.

Hydroxyl radical scavenging activity (%) = [1 − (A_1_ − A_2_)/A_0_] × 100%

A_0_: absorbance value of sample solution replaced by distilled water;

A_1_: absorbance measured at 510 nm;

A_2_: absorbance value of salicylic acid–ethanol solution replaced by anhydrous ethanol.

### 2.8. DPPH Radical Scavenging Activity

The evaluation of DPPH radical scavenging potential was undertaken following the protocol of Nunes et al. [34]. A combination of 2 mL of MPs solution and 2 mL of a 0.2 mmol/L DPPH solution was prepared and subsequently incubated in darkness at a temperature of 25 °C for 30 min. After incubation, the absorbance of the mixture was quantified at a wavelength of 517 nm employing a UV2000 spectrophotometer. To establish a baseline, the control group was replaced with methanol. The quantification of DPPH scavenging efficacy was computed using the ensuing mathematical expression. Each distinct sample was subjected to analysis in triplicate.

DPPH scavenging activity (%) = [1 − (A_i_ − A_j_)/A_0_] × 100%

A_0_: absorbance value of a mixture of water and DPPH solution at 517 nm;

A_i_: absorbance value of a mixture of sample solution and DPPH solution at 517 nm;

A_j_: absorbance values of a mixture of sample solution and methanol at 517 nm.

### 2.9. Differential Scanning Calorimetry (DSC)

The investigation into the thermal denaturation behavior of the protein within the MPs specimens was conducted employing a differential scanning calorimeter (Hesheng HS-DSC-101, Shanghai, China), as detailed in reference [35]. The experimental procedure involved a controlled heating process, utilizing a heating rate of 10 °C/min and encompassing a temperature range from 20 °C to 120 °C. The resulting denaturation temperatures (Td, °C) and corresponding heat absorption values (J g^−1^) were meticulously quantified. In order to ensure robustness and precision, each distinct sample was analyzed three times.

### 2.10. Particle Size

Particle size analysis was conducted employing a protocol adapted marginally [36]. In this method, a 1.5 mL aliquot was subjected to centrifugation at 8819 g for 10 min. Subsequently, the particle size distribution pertinent to the MPs within the specimen was determined using a nanoparticle size analyzer (Malvern 2000, Malvern City, UK) operating under a controlled temperature of 25 °C. It is imperative to underscore the meticulous rigor of the analysis, with each individual sample being analyzed in triplicate to ensure reliability and statistical significance.

### 2.11. Molecular Fluorescence

The quantification of molecular fluorescence was conducted in accordance with the methodology outlined by Huang et al., with a slight modification [37]. The sample solution, initially prepared at a concentration of 1 mg/mL, was subsequently diluted to 0.5 mg/mL using a 0.6 mol/L NaCl solution. The ensuing analysis involved the utilization of a fluorescence spectrophotometer (Hitachi F-7000, Tokyo, Japan). Notably, the instrumental parameters encompassed an excitation wavelength of 296 nm, an excitation spacing set at 2.5 nm, an equivalent emission spacing of 2.5 nm, a data collection rate of 12,000 nm/min, and spectral recording spanning the range of 270–350 nm. It should be noted that each distinct sample was analyzed in triplicate in order to establish reproducibility and statistical significance.

### 2.12. Statistical Analysis

All experiments were conducted independently and in triplicate using MPs from three sample preparations. Experimental data were presented in the form of the mean ± standard deviation (SD). A two-way analysis of variance (ANOVA) was performed to determine statistical significance using SPSS statistical software (version 19.0, Chicago, IL, USA). The visualization of data and its systematic tabulation were facilitated by leveraging the capabilities of OriginPro (2021) 9.8 software. In order to discern and elucidate notable disparities among means, the methodology of Duncan’s multiple range tests was harnessed, with a predetermined significance threshold of 0.05.

## 3. Results and Discussion

### 3.1. Carbonyl and Free Sulfhydryl Group Content

**Figure 1:**
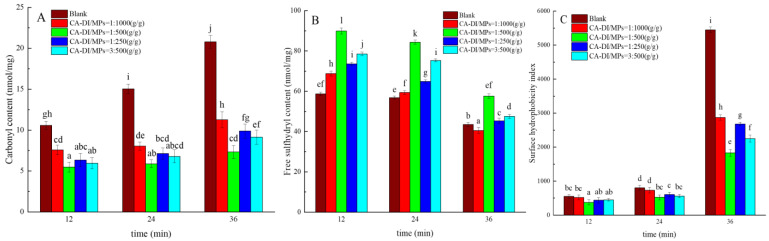
Effects of capsaicin (CA) and dihydrocapsaicin (DI) on carbonyl content (**A**), free sulfhydryl content (**B**), and surface hydrophobicity (**C**) of myofibrillar proteins (MPs) during heat treatment. The label ratios (1:1000, 1:500, 1:250, and 3:500) in the graphs represent the CA–DI to MPs ratio (g/g), and the blank group refers to MPs without CA–DI addition. Different lowercase letters in the graph indicate significant differences among the 15 treatment means (*p* < 0.05).

The quantification of carbonyl group and free sulfhydryl group content within proteins is a pivotal marker for assessing protein oxidation dynamics, as determined by previous studies [38,39]. As illustrated in Figure 1A,B, with the prolongation of heat treatment, MPs of the control group exhibited a significant increase in the carbonyl content and a decrease in the free sulfhydryl groups (*p* < 0.05). The formation of carbonyl compounds and the loss of sulfhydryl groups are some of the most common expressions of the chemical damage to oxidized proteins. This is consistent with the findings of Valquíria et al. [40]. The degree of MP’s oxidation escalated in tandem with prolonged heat treatment. The escalation in oxidative stress at elevated temperatures precipitated diverse physicochemical alterations, including cellular compartmental disarray, the liberation of catalytic iron, and the generation and cleavage of hydroperoxides, which collectively promoted a heightened incidence of protein carbonylation [41].

A significantly notable mitigation of protein oxidation occurred, as evidenced by the reduced carbonyl group content, across all experimental groups incorporating CA–DI supplementation compared with the control group (*p* < 0.05). At a ratio of 1:500 (CA–DI to MPs, g/g) within a 12 min timeframe, the carbonyl content declined by 64.77%, while free sulfhydryl content increased by 32.53%. CA–DI is fat-soluble, whereas MPs are amphiphilic macromolecules [28]. The addition of excess CA–DI might affect its combination with MPs in the complex blending system. The excess hydrophobic bonds of CA–DI exposed in the solutions result in aggregation of CA–DI inhibiting the blending. It was within this timeframe that the extent of MP oxidation reached its nadir. Furthermore, it is noteworthy to mention that, as the duration of heat treatment increased, the carbonyl content within MPs was augmented, whereas the free sulfhydryl content decreased. This sequence aligns with the observations made by Bao et al., where heightened oxidative levels were accompanied by the concurrent loss of free sulfhydryl groups and histidine, coupled with enhanced carbonyl formation within myofibrillar structures [42]. These findings substantiate the congruence of our study with the investigations by Bu et al. [43].

### 3.2. Surface Hydrophobicity

Figure 1C illustrates the surface hydrophobicity variations of MPs across distinct ratios of CA–DI under different heat treatment durations. A conspicuous augmentation in hydrophobicity was discerned in the blank MPs as the duration of heat treatment increased, culminating in a hydrophobicity index of 5450 after 36 min, with an increase of 197.81% compared with the 12 min treatment period. Analogous observations were made by Morzel [44] on heat-treated skeletal muscle MPs. This phenomenon can be attributed to the susceptibility of hydrophobic regions within MPs to degrade at elevated temperatures, thereby exposing more hydrophobic moieties to the polar milieu [45]. Moreover, heightened temperatures are conducive to oxidative processes, triggering intermolecular aggregation and crosslinking, ultimately bolstering surface hydrophobicity [46].

In contrast to the untreated group, the surface hydrophobicity of MPs exhibited a significant reduction in all capsaicin–dihydrocapsaicin treatment groups (*p* < 0.05). In this configuration, the surface hydrophobicity of MPs decreased by 50.55% compared with the untreated group upon 36 min of heat treatment. High temperatures in meat processing can oxidize proteins and lead to aggregation and crosslinking between the molecules, enhancing their surface hydrophobicity [47]. This substantiates the inference that the incorporation of CA–DI inhibits the side chain carbonylation of amino acids and the formation of polypeptide chain crosslinks within MPs. Conversely, elevated proportions of CA–DI beyond the 1:500 (g/g) threshold yielded a contrasting outcome, precipitating the degradation of MP structure and concomitant escalation of surface hydrophobicity. A high concentration of lipophobic CA–DI might bring aggregation because of its limitary solubility in complex solutions. When the ratio of CA–DI to MPs was over 1:500, the degree of the blending system was suppressed, and the corresponding surface hydrophobicity decreased. This result agrees with the fluctuations observed in the carbonyl and free sulfhydryl group content.

### 3.3. •OH and DPPH Scavenging Rates

**Figure 2:**
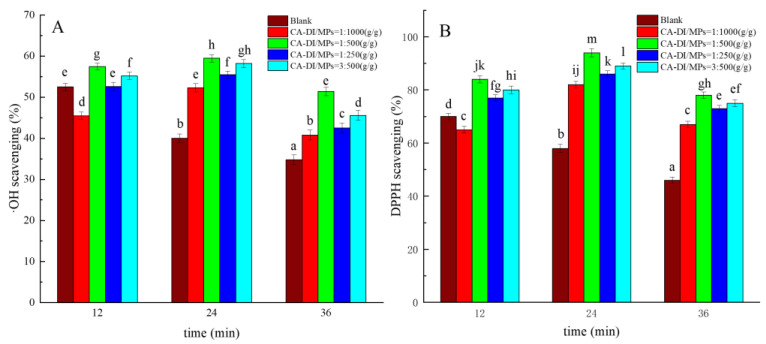
Effects of capsaicin (CA) and dihydrocapsaicin (DI) on •OH-free radical scavenging rate (**A**) and DPPH-free radical scavenging rate (**B**) of myofibrillar proteins (MPs) during heat treatment. The label ratios (1:1000, 1:500, 1:250, and 3:250) in the graphs indicate CA–DI to MPs (g/g) and the blank group indicates MPs without CA–DI addition. Different lowercase letters in the graph indicate significant differences among the 15 treatment means (*p* < 0.05).

Elevated thermal conditions have been observed to readily trigger the generation of hydroxyl (OH) radicals and 2,2-diphenyl-1-picrylhydrazyl (DPPH) radicals within meat products, thereby causing oxidative deterioration in proteins and the consequent protein degradation, thereby impairing the quality attributes of meats [48]. As portrayed in Figure 2A,B, the •OH and DPPH radical scavenging rates within the control group were notably reduced after 24 min of heat exposure compared to the 12 min interval (*p* < 0.05). A significant reduction for each concentration was observed in scavenging efficacy between the 24 min and 36 min intervals (*p* > 0.05). Notably, when the CA–DI to MPs ratio was maintained at 1:500 (g/g) over 24 min of heat treatment, MPs demonstrated their highest recorded scavenging rates for •OH and DPPH radicals, reaching 59.5% and 94.0%, respectively. This exceptional radical scavenging rate of MPs at this specific ratio can be attributed to the proper incorporation of an optimal concentration of CA–DI. This might be attributed to the excess content of CA–DI, which could not combine with the MPs, resulting in a reduced radical scavenging rate when the concentration was increased to 1:250 and 3:500 (CA–DI, g/g). This finding aligns with the observations that 1% clove extract exhibited elevated DPPH radical scavenging potential in comparison to the control group [49]. Correspondingly, Muppalla et al. demonstrated the efficacy of 1% seed cover extract from Zanthoxylum rhetsa in extending the shelf life of chicken meat via its antioxidative effects [50].

### 3.4. DSC Analysis

**Figure 3:**
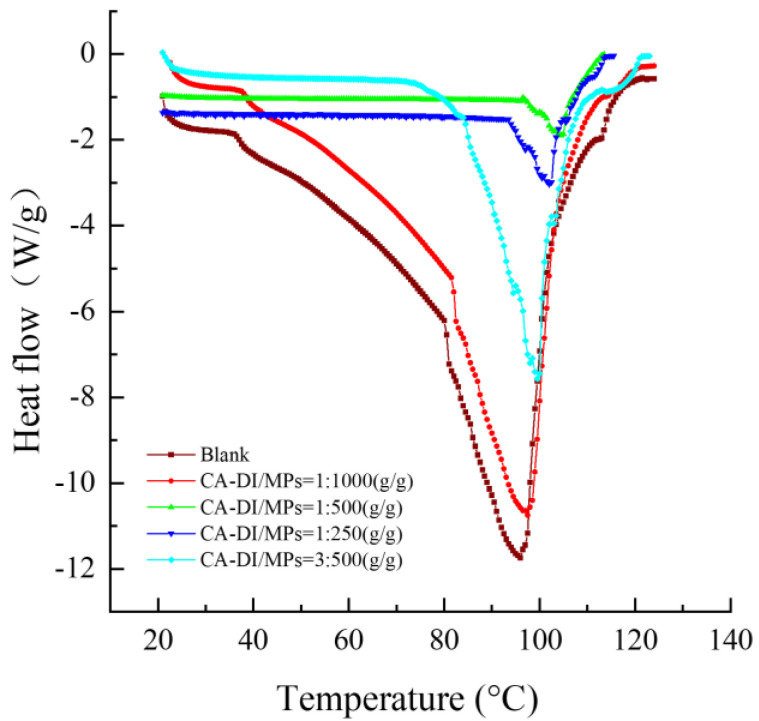
DSC of myofibrillar proteins with different capsaicin and dihydrocapsaicin content. The label ratios (1:1000, 1:500, 1:250, and 3:500) in the graphs represent the CA–DI to MPs (g/g) and the blank group indicates MPs without CA–DI addition.

The investigation of meat denaturation temperature, a critical indicator of protein thermal stability during heat processing [51], was undertaken utilizing differential scanning calorimetry (DSC), as detailed in reference [52]. From the data presented in Table 1 and depicted in Figure 3, the initial denaturation temperature exhibited a notable increase across all groups subjected to the CA–DI treatment, relative to the control group. The apical point of initial denaturation temperature reached 96.62 °C in the context of a CA–DI to MPs ratio of 1:500 (g/g). Similar observations were made in the context of the peak denaturation temperature, which culminated at 103.04 °C when employing the same 1:500 (CA–DI to MPs, g/g) ratio. This phenomenon is attributed to the propensity of capsaicin and dihydrocapsaicin to engage in substantial interactions with myofibrillar proteins, which consequently confers enhanced thermal stability. A comparable study by Su et al. illustrated analogous outcomes, wherein rabbit myofibrillar proteins exhibited heightened thermal stability subsequent to conjugation with quercetin [53].

Observations from Table 1 showed the reduction in heat absorption within the CA–DI treated groups, with the largest heat absorption recorded at 200.24 J·g^−1^ in instances wherein the CA–DI to MPs ratio was maintained at 1:500 (g/g). From these findings, it is inferred that under this specific ratio, the thermal denaturation of myofibrillar proteins is minimized. Protein oxidation causes the formation of protein cross-links by covalent bonding (hydrogen bonding) within a protein or between proteins [54,55]. Optimal levels of CA–DI are posited to mitigate heat absorption by meat proteins and curtail intramolecular covalent bond cleavage, thereby concomitantly augmenting the denaturation temperature and bolstering thermal stability. A complementary study by Chen et al. underscored the inhibitory effect of clove extract on protein oxidative denaturation by virtue of diminished heat absorption during thermal treatment [56]. Nonetheless, an excessive concentration of CA–DI may precipitate the disruption of chemical bonds within meat proteins, thereby exerting a negative impact on their thermal stability.

### 3.5. Particle Size

**Figure 4:**
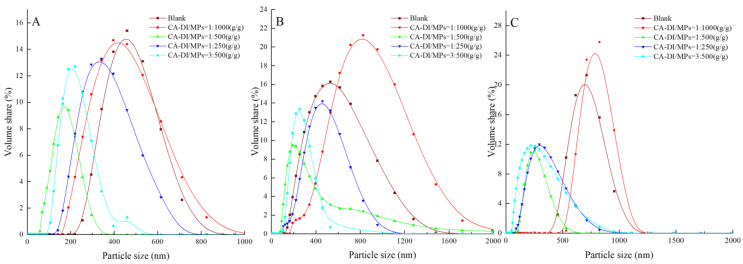
Effects of capsaicin and dihydrocapsaicin on the particle size of myofibrillar proteins heated for 12 min (**A**), 24 min (**B**), and 36 min (**C**). The label ratios (1:1000, 1:500, 1:250, and 3:500) in the graphs indicate the CA–DI to MPs (g/g) ratios and the blank group refers to MPs without added CA–DI.

The analysis of myofibrillar protein particle size serves as a pivotal indicator elucidating the extent of protein cross-linking consequent to thermal processing, thereby having direct implications on the texture properties of duck meat [57]. The elucidation of the particle size varying by the CA–DI to MPs ratios is shown in Figure 4. In the absence of CA–DI supplementation, the curve of MP particle size tended to gravitate towards an augmented particle size. This observation indicates the potential denaturation-induced aggregation of MPs and the concomitant development of protein cross-links upon exposure to thermal treatment, which culminate in increased particle size of MPs. An instructive account by Huang et al. corroborates this finding, outlining the propensity for heightened temperatures to precipitate the perturbation of internal hydrogen bonds within MPs, thereby causing the unfolding of the MPs macromolecules [58].

Conversely, the addition of CA–DI caused a reduction in particle size in comparison to the control, apart from the 1:1000 CA–DI to MPs (g/g) ratio. The particle size of MPs was minimal in samples at a ratio of 1:500 (CA–DI to MPs, g/g) in 12 min heat treatment. This empirical revelation underscores the substantive capacity of CA–DI to effectively counteract protein aggregation dynamics, thereby providing increased control over protein particle size distribution. However, when the CA–DI to MPs ratio surpassed 1:500 (g/g), it caused the enlargement of myofibrillar protein particle size compared to the control group. This tendency is ascribed to the potential accentuation of hydrophobic interactions and the corresponding augmentation of the degree of protein polypeptide chain cross-linking. Drawing a parallel, the observations of Zhang et al. reveal analogous tendencies in the context of curcumin supplementation, highlighting that the measured augmentation of curcumin concentrations (ranging from 40 to 80 μg/mL) engendered heightened stability, while an excessive curcumin dosage precipitated the formation of considerable protein aggregates [59].

### 3.6. Endogenous Amino Acids

**Figure 5:**
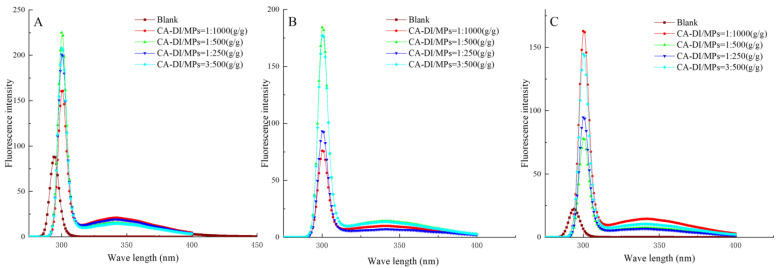
Effects of capsaicin and dihydrocapsaicin on the endogenous amino acids of myofibrillar proteins heated for 12 min (**A**), 24 min (**B**), and 36 min (**C**). The labeled ratios (1:1000, 1:500, 1:250, and 3:500) in the graphs indicate the CA–DI to MPs (g/g) ratio, and the blank group refers to MPs without CA–DI addition.

The evaluation of tertiary structural alterations in heat-processed MPs can be performed using fluorescence spectroscopy to analyze the fluorescence intensity of endogenous amino acids [60]. Figure 5 illustrates the endogenous fluorescence spectra inherent to the complete spectrum of examined MPs. Notably, the fluorescence emission peaks for experimental groups were recorded close to 300 nm, whereas those for the control counterparts were at a 296 nm wavelength, thus delineating a red shift across all CA–DI-treated samples. This characteristic spectral perturbation increases our conjecture regarding the enhanced hydrophobicity resident within amino acid moieties contained with the MPs. The fluorescence intensity attained a maximum of 221.7 when the ratio of CA–DI to MPs and heat treatment time were 1:500 (g/g) and 12 min, respectively. Pertinent literature postulates that aromatic amino acid residues (*Trp* and *Tyr*) conventionally reside within the hydrophobic core of proteins; nevertheless, underpinning the conformational changes arising from denaturation or ligand interactions may expose these residues to the ambient milieu [40,61]. It is proposed that within the test groups, CA–DI potentially engenders interaction with certain amino acid moieties, ostensibly mitigating chemical bond cleavage during the MPs unfolding induced by thermal treatment, in contrast to the control group. Nonetheless, protracted thermal exposure still invariably contributes to the exposition of solvent-accessible chromophores, resulting in lower fluorescence intensity across all groups. This phenomenon agrees with existing investigations [62].

## 4. Conclusions

The addition of CA–DI into the heat treatment paradigm has a remarkable influence on protein structure via complex interactions with MPs, thereby affecting physicochemical attributes and altering the functional properties of the protein. Notably, the addition of CA–DI mitigated the formation of carbonyl groups and protected the sulfhydryl groups in MPs of duck meat. In addition, CA–DI reduced the surface hydrophobicity of protein and promoted the free radical scavenging rate during the heat treatment within MPs. DSC analyses showed that CA–DI increased the thermal denaturation temperature while concomitantly enhancing the overall thermal stability of MPs. It has also been found that the combination of CA–DI with endogenous amino acids is critical to maintaining the protein particle size of MPs. However, caution is warranted with regard to excessive CA–DI supplementation, and prolonged thermal exposure may culminate in the erosion of essential functional characteristics of MPs. Consequently, it is important to control the dose of CA–DI during the processing of meat products to inhibit the oxidation of meat products. The proper use of CA-DI could also enhance the thermal stability of duck meat and contribute to the meat quality. The objective of this study was to provide a reference for investigating the effect of capsicum on duck meat protein properties during heat treatment. To further clarify the mechanism, it is necessary to conduct more research into the changes in protein conformation, particularly amino acids, the different types of chemical bonds, and their related characteristics during the heat processing of meat.

## Figures and Tables

**Table 1:** Effects of different capsaicin (CA) and dihydrocapsaicin (DI) concentrations (blank, 1:1000, 1:500, 2:500, and 3:500, g/g) on the denaturation temperature and heat absorption of myofibrillar proteins (MPs). Reported results correspond to mean ± standard deviation. Different letters within the same column indicate significant differences (*p* < 0.05).

Ratio of CA–DI to MPs (g/g)	Initial Denaturation Temperature T_d_ (°C)	Heat Absorption (J g ^−1^)	Peak Denaturation Temperature (°C)
Blank group	69.93 ± 0.35 ^a^	1825.00 ± 22.17 ^e^	93.85 ± 1.49 ^a^
1:1000	77.92 ± 1.14 ^b^	1714.00 ± 24.30 ^d^	97.27 ± 1.04 ^ab^
1:500	96.62 ± 1.35 ^d^	200.24 ± 5.03 ^a^	103.04 ± 1.48 ^c^
2:500	94.26 ± 1.09 ^d^	314.42 ± 7.54 ^b^	101.68 ± 1.63 ^bc^
3:500	84.47 ± 0.64 ^c^	765.34 ± 12.82 ^c^	98.35 ± 0.85 ^b^

**Figure A1:**
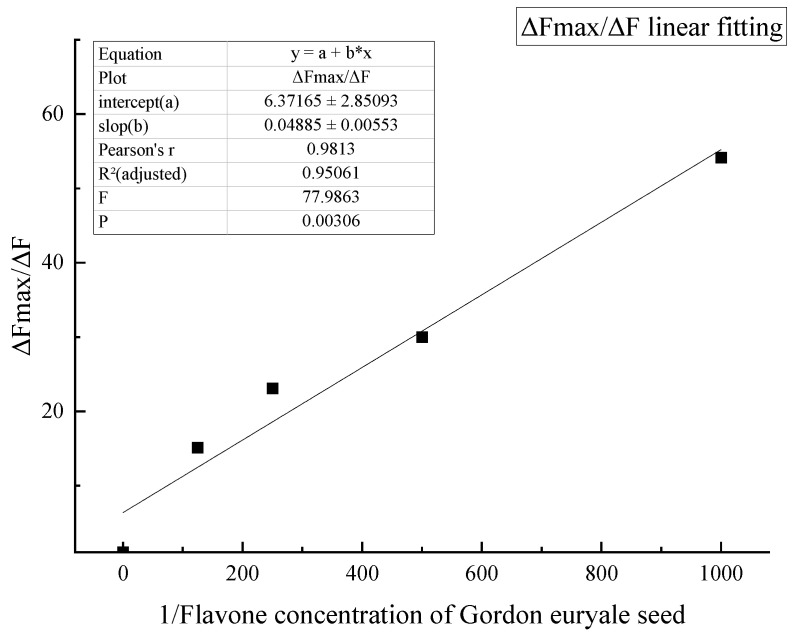
The Bennich-Hildebrand diagram of the combination of capsaicin with dihydrocapsaicin (CA–DI) and myofibrillar proteins.

## Data Availability

Data are contained within the article.

## Appendix A

As shown in Figure A1, it can be seen that, during the heat treatment, CA–DI and MPs were measured at ratios of 1:1000, 1:500, 1:250, 3:500 (g/g), i.e., CA–DI mass concentrations of 1/c (125, 250, 500, 1000 (g/L)^−1^), 1/c and the corresponding ΔFmax/ΔF (15, 23, 30, 54) all satisfied the Benesi-Hildebrand equation: ΔFmax/ΔF = 1 + 1/c, (The fitted equation: y = 6.37165 + 0.04885∗x, R² = 0.965061, *P* = 0.00306 ≤ 0.01, it means statistical extremely significant) indicating that CA–DI reacted with MPs at the above mass ratios and the binding mechanism is dynamic. CA–DI was employed in MPs at ratios of 1:1000, 1:500, 1:250 and 3:500 (g/g).
